# Exploring the Effects of Graphene-Based Nanoparticles on Early Salmonids Cardiorespiratory Responses, Swimming and Nesting Behavior

**DOI:** 10.3390/jox14020029

**Published:** 2024-04-14

**Authors:** Tomas Makaras, Magdalena Jakubowska-Lehrmann, Živilė Jurgelėnė, Sergej Šemčuk

**Affiliations:** 1 Nature Research Centre, Akademijos St. 2, 08412 Vilnius, Lithuania; zivile.jurgelene@gamtc.lt; 2 National Marine Fisheries Research Institute, Kołłątaja St. 1, 81-332 Gdynia, Poland; mjakubowska@mir.gdynia.pl; 3 Center for Physical Sciences and Technology, Saulėtekio Av. 3, 02300 Vilnius, Lithuania; sergej.semcuk@ftmc.lt

**Keywords:** gill ventilation, heart rate, larval stage, juveniles, nests, swimming activity

## Abstract

Graphene-based nanomaterials are exceptionally attractive for a wide range of applications, raising the likelihood of the release of graphene-containing nanoparticles into aquatic environments. The growing use of these carbon nanomaterials in different industries highlights the crucial need to investigate their environmental impact and evaluate potential risks to living organisms. The current investigation evaluated the nanotoxicity of graphene (nanoflakes) and graphene oxide (GO) nanoparticles on the cardiorespiratory responses (heart rate, gill ventilation frequency), as well as the swimming and nesting behavioral parameters of early stage larvae and juvenile salmonids. Both short-term (96 h) and long-term (23 days) exposure experiments were conducted using two common species: brown trout (*Salmo trutta*) and rainbow trout (*Oncorhynchus mykiss*). The findings demonstrated notable alterations in fish nesting behavior, swimming performance, and cardiorespiratory functions, indicating the potential toxicity of nanoparticles. This impact was observed at both physiological and whole-organismal levels in salmonids at early stages. Future investigations should explore different types of nanocarbons and their potential enduring effects on fish population structure, considering not only individual survival but also broader aspects of development, including feeding, reproductive, and other social dynamics.

## 1. Introduction

Carbon nanomaterials, including graphene, which have been discovered in the last several years, exhibit remarkable properties with significant potential across a spectrum of applications [1,2]. These applications encompass energy storage, electronics, nanocomposites, medical applications, extreme condition systems, desalination, antifouling agents, and the purification of pollutants from seawater. The demand for such nanocarbons is expected to exceed 1 kiloton annually by the early 2025s, with additional growth foreseen as the nanotechnology sector progresses [3]. The increasing utilization of nanocarbons, combined with the lack of regulatory constraints and efficient waste management, presents a growing threat to both the environment and human safety [4]. Therefore, it is essential to meticulously define and establish scientifically valid thresholds for the presence of nanomaterials in aquatic environments, ensuring the sustainability and welfare of aquatic species by considering factors such as size, composition, and concentration.

The existing or anticipated environmental concentrations of carbon nanomaterials are unclear because of inadequate measurement methods. Due to limitations in analytical methodologies, the fate of these nanomaterials in the environment remains unknown.

While the technological processes for synthesizing various graphene-based nanomaterials (GBNs) may be similar, slight variations in the properties of the final products can lead to diverse induced toxicity effects. Thus, when identifying adverse effects on aquatic biota from a particular material, it is essential to comprehensively understand not only the carbon nanomaterials themselves but also to attribute these effects to their physicochemical characteristics. The toxicity of GBNs might be influenced by specific characteristics such as linear size, particle form, quantity of layers, surface characteristics, functionalization, impurities, and their susceptibility for aggregation, clustering, and sedimentation [5,6]. The dynamic interaction of GBNs with living organisms, spanning a size range from a few to 100 nm, stands as a critical factor influencing their compatibility within biological systems [7]. Whether taken in by organisms or adhering through biological barriers such as skin, mucosal membranes, and the blood–brain barrier, GBNs harbor the potential to instigate mechanical damage to cells, thereby disrupting diverse cellular functions [8]. These disturbances extend to the induction of oxidative stress, the alteration of cell viability, and the modulation of immunological, physiological, and behavioral responses, collectively impacting the overall health of the organism [9,10,11].

Recent studies have revealed that graphene oxide (GO) can induce genotoxic, immunotoxic, and cytotoxic changes in living organisms, including enhanced apoptosis, oxidative stress, cardiac and metabolic impairments, and disruptions in fish embryo development, all of which have implications for their survival and population structure [12,13,14,15]. The toxic mechanisms of GO-induced toxicity in fish have also been extensively examined at the cellular and molecular levels [16,17]. However, most of the studies demonstrating the adverse effects in fish focus on the crucial role that fish play in the transport of nanomaterials within and beyond water body boundaries. The success of population recruitment is heavily dependent on the well-being of these early life stages. Many of these studies were primarily conducted in vivo using zebrafish (*Danio rerio*) [12,13,18,19], or a Japanese medaka (*Oryzias latipes*) which are known as a model species characterized by a relatively brief embryonic and larval development [20,21]. Nevertheless, the impact of carbon nanomaterials on the initial phases of different fish species, such as salmonids, which undergo an extended embryonic development, remains uncertain. While salmonids are an economically valuable fish species that naturally inhabit aquatic environments and may be directly exposed to various nanocarbons, there is a scarcity of toxicological data pertaining to these species. Information on induced effects at different stages of development and across various biological levels is lacking. Therefore, further exploration is warranted across a spectrum of scales, from cellular to whole-organism levels.

To enhance understanding and supplement existing data on the effects of GBNs on freshwater salmonids, this study examined the short-term (4 days) and long-term (23 days) impacts of graphene (nanoflakes) and GO on brown trout (*Salmo trutta*) and rainbow trout (*Oncorhynchus mykiss*) at various developmental stages (larvae and juveniles). Utilizing the popular form of graphene (nanoflakes) and the more economical and extensively studied GO as its precursor, we assessed physiological parameters such as heart rate (HR) and gill ventilation frequency (GVF), along with nesting rate and swimming activity. These analyses aimed to elucidate the influence of these carbon nanomaterials at both physiological and whole-organismal levels, providing insights into early stage fish behavioral traits that could impact their survival.

## 2. Materials and Methods

### 2.1. Study Animal

Brown trout (*S. trutta*) and rainbow trout (*O. mykiss*) eyed-stage embryos were obtained from fish hatcheries in Trakų Vokė, Lithuania, and Dąbie, Poland. Rainbow trout embryos followed the incubation protocol as described in [22]. In 1.7 L glass containers, 300 rainbow trout eggs were placed, along with 1.5 L of freshwater (filtered through GF/F filters; Whatman 47 mm, 0.7 μm) and 200 g of gravel sediment (3 mm in diameter). These containers were incorporated into a closed-loop recirculation system equipped with a cooling unit (Titan 2000, Aqua Medic, Bissendorf, Germany) to maintain a steady water temperature of 9 ± 0.5 °C.

Brown trout eggs were put in incubators (430 × 510 × 150 mm) made of porous stainless steel (2 mm in diameter), ensuring a steady water flow at a temperature of 8 ± 0.5 °C. Brown trout juveniles, originating from a stock population within the same spawning, were accommodated in Recirculating Aquaculture Systems (RAS) in 1 m^−3^ capacity tanks. The flow-through system, with a water density below 25 kg m^−3^, maintained a flow rate of 10–15 mm s^−1^, and the environment was controlled at a pH of 8 and a temperature of 12 °C, following natural dark–light cycles.

Experimental trials were conducted on larval stages (1-day post-hatching) for both *S. trutta* and *O. mykiss*, as well as on *S. trutta* juveniles (~8 months old, 0+) with a weight (mean ± SD) of 5.91 ± 1.42 g and a length of 6.51 ± 0.72 cm (*n* = 80).

All biological testing followed the guidelines specified in [23] for the ethical treatment of animals used in scientific research. The protocol received approval from the Animal Ethics Committees of both the Lithuanian State Food Veterinary Service (license no. G2-168; valid from 8 February 2021 to 7 February 2025).

### 2.2. Synthesis and Characterization of Graphene Oxide

The fresh portion of GO was synthesized based on methodology described in our previous study [24]. The synthesized GO was subjected to thorough characterization via Fourier-transform infrared spectroscopy (FTIR), X-ray powder diffraction (XRD), Raman spectroscopy, thermal gravimetric analysis (TGA), X-ray fluorescence (XRF), and scanning electron microscopy (SEM) analysis.

**Figure 1:**
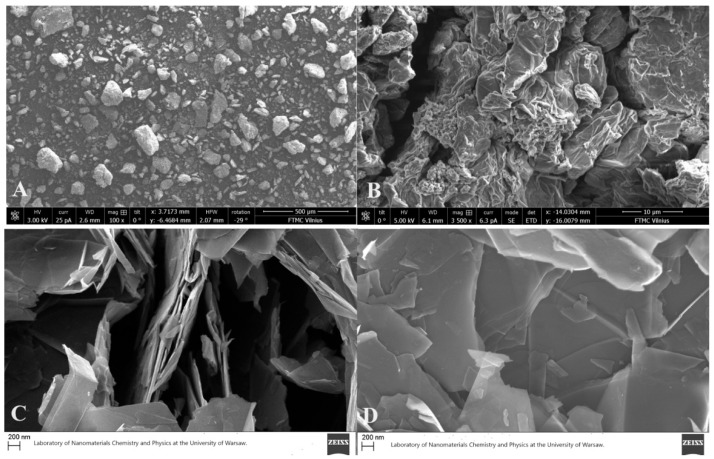
SEM images depicting the synthesized graphene oxide (GO) (**A**,**B**), and graphene (nanoflakes) (**C**,**D**). SEM images adapted from our previous studies [24,27].

The FTIR spectra of prepared GO were recorded with ALPHA spectrometer (Bruker Inc., Ettlingen, Germany) from 400 to 3800 cm^−1^ with resolution of 4 cm^−1^ to affirm the formation of functional groups: carboxyl, epoxy, and hydroxyl. The recorded spectra exhibited peaks at 1053, 1080, 1230 1420, 1621, 1736 cm^−1^ and one wide 3000–3300 cm^−1^, corresponding to C–O, C–O–C, C–OH, C–C, C=O, and O–H vibrations, respectively. The Raman spectra was recorded using RAM II system (Bruker Inc., Ettlingen, Germany) with a 1064 nm laser. The D ang G bands were detected at ranges 1300–1350 cm^−1^ and 1595–1610 cm^−1^, respectively. The calculated intensity ratio of those bands was 0.76, indicating a high degree of oxidation of synthesized GO [25]. The XRD pattern was captured utilizing a RIGAKU X-ray diffractometer from Japan and provided for analysis with the PDXL software package (version 2.8.4.0). The successful synthesis of GO was validated by a significant peak at 2θ = 10.44°. Moreover, calculations of crystallite size of GO applying the Halder–Wagner method [26] showed a result of 6.4 ± 0.4 nm. The purity of produced GO was examined with TGA (STA Pt 1600 with mass spectrometer Thermostar GDS 320, Linseis, Selb, Germany) and XRF (Fluorescent X-ray spectrometer, Panalytical, Almelo, The Netherlands) techniques. Both detected the sulphur as a main impurity (potentially originating from the H_2_SO_4_ used during the synthesis) with 0.119% of tested samples and a purity of GO was 99.621 ± 0.011%. The size distribution was examined with SEM Helios NanoLab 650 (FEI, Eindhoven, The Netherlands). Analysis of images showed that the size of synthesized GO sheets is, on average, 10 µm, with nm scale thickness (Figure 1A,B).

The commercially available graphene (nanoflakes) were purchased from Graphene Supermarket Inc. (Ronkonkoma, New York, NY, USA). Because of natural aggregation, the thickness might range from one to a few graphitic sheets. The linear size of these sheets fluctuates between 8 and 20 nm, with a grain size distribution approximately ~12 nm (Figure 1C,D).

### 2.3. Experimental Design

**Figure 2:**
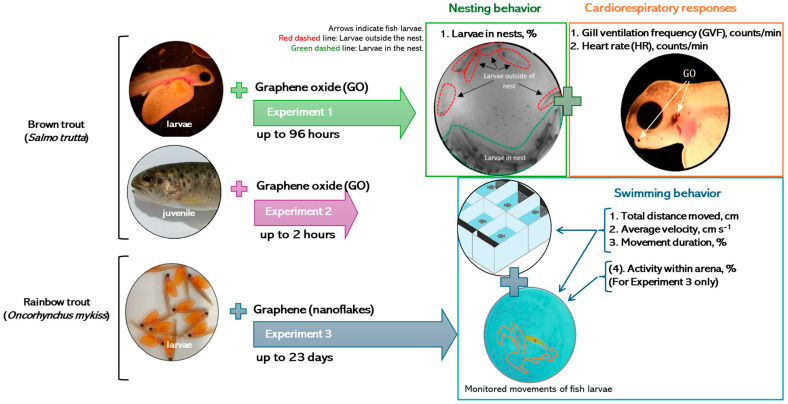
A flowchart of experimental settings.

To analyze the impact of graphene (nanoflakes) and GO on brown trout (*S. trutta*) and rainbow trout (*O. mykiss*), the research was divided into short-term (up to 96 h) and long-term (up to 23 days) experiments, considering the stage of fish development and the tested parameters. A schematic representation of the experimental settings is provided in Figure 2.

#### 2.3.1. Experiment 1: Short-Term Exposure of *S. trutta* Larvae to GO

Short-term behavioral tests were conducted on *S. trutta* larvae and juveniles, exposing them to various concentrations of GO (0, 1.0, 10.0, 20.0, and 40.0 mg L^−1^). Fish larvae were tested in groups of 10 per replicate (40 per treatment) in 0.6 L glass beakers filled with 0.5 L of water with dispersed GO achieved through stirring or ultrasonication with EMAG EM40HC (at 38 kHz) for up to 2 min period. The control group received GO-free water from RAS.

Experiments lasted 96 h in a climate chamber (PGC-660, Bronson, Zaltbommel, The Netherlands) at a constant water temperature of 8 ± 0.5 °C and under dark lighting conditions. Continuous aeration was maintained to maximize oxygen saturation and minimize GO particle aggregation intensity.

The quantity (expressed as a percentage, %) of larvae within nests (identified when one larva closely interacts with another) was observed and computed after 24, 48, 72, and 96 h of exposure. Heart rate (HR) and gill ventilation frequency (GVF) of 5 larvae per treatment were calculated after 96 h. Cardiorespiratory monitoring was carried out after 96 h of exposure, examining each larva for 15 s in the dark to avoid light interference. No deceased larvae were observed throughout the 96 h exposure period.

Experiments with larvae were conducted in compliance with ISO standards [28] and the OECD recommendations for acute toxicity tests [29].

#### 2.3.2. Experiment 2: Short-Term Exposure of *S. trutta* Juveniles to GO

For the analysis of swimming activity, individual *S. trutta* juveniles were placed in open-field glass tanks (350 × 200 × 200 mm), with 16 individuals per treatment. Each tank was filled with 3 L of water (13 ± 1 °C) sourced from RAS. Fish acclimatization lasted 30 min prior to testing. In exposure trials, the GO test concentration, similar to that used for exposed fish larvae, was introduced and thoroughly mixed to prevent particle aggregation.

The trials were carried out under static water conditions, signifying that the water remained unchanged until the experiment’s conclusion. The water was consistently aerated. The temperatures of the water and dissolved oxygen (DO) saturation (99.7%) in the experimental tanks holding the test fish were steady throughout the study.

Swimming activity data were analyzed using Ethovision XT 16 (Noldus Inc., Wageningen, The Netherlands) video tracking software. Common endpoints such as total distance moved (cm), average velocity (cm s^−1^), and cumulative movement duration (%) were selected for analysis. Video data were gathered at intervals of 10 min throughout the 2 h experimental periods. Fish swimming behavior was evaluated by consolidating group-mean data after a 2 h period.

Experiments 1 and 2 were carried out at the Nature Research Centre (Lithuania).

#### 2.3.3. Experiment 3: Long-Term Exposure of *O. mykiss* Larvae to Graphene (Nanoflakes)

This study examined the prolonged impact (23 days) of graphene (nanoflakes) on the swimming behavior of *O. mykiss* larvae (220 D°) in accordance with a previous study [29]. Each individual larva was placed in transparent, cylinder-shaped plastic jar (70 × 100 mm) filled with 0.3 L of a graphene (nanoflakes) solution at a concentration of 4 mg L^−1^, consistent with the exposure groups used for stocking (5 larvae each replication, total of 15 per treatment). Additionally, larval groups (10 individuals per replicate, 90 in total per treatment) were exposed, and their overall group activity duration, measured as pixel changes across the observational arena (activity within arena, %), was assessed using the same video tracking software. All larvae were simultaneously monitored in their respective jars.

The choice of the 4 mg L^−1^ concentration was based on the outcomes observed in a short-term experiment and the observed effects at the lowest concentration of graphene oxide (GO). It also aligns with the lower range of carbon nanomaterial concentrations commonly applied in experimental studies on fish’s early life stages [12,15,30,31].

The jars were moved to a flow-through system located in an open-field glass tank to ensure a stable water temperature (9 ± 1 °C) and monitor the movement of the larvae. A 30 min acclimation period preceded the recording of swimming activity. Trials were carried out under static water conditions and natural lighting, with dissolved oxygen (DO) levels consistently maintained above 9.6 mg L^−1^. Data were gathered at 10 min intervals during a 5 min recording period, and group-mean data after 1 h were employed for additional analysis, as outlined in Section 2.4.

Experiment 3 was carried out at the National Marine Fisheries Research Institute (Poland).

### 2.4. Statistical Analysis

Statistical data evaluation included checking for normality using the Shapiro–Wilk test and assessing homoscedasticity through the Levene test. Visual inspection of residuals was conducted, and when necessary, log- or square-root transformations were applied, followed by re-analysis to meet parametric assumptions. If the requirements were not met post-transformation, non-parametric tests were employed.

Nesting and short-term swimming behavioral data over time exposure underwent Repeated Measures ANOVA, with Mauchly’s sphericity test ensuring model fitness. As no significant RM ANOVA model, including the time variable, was identified, the swimming activity data over time was consolidated and subjected to one-way ANOVA. Fish larval swimming data over 16-, 20-, and 23-day periods were analyzed using linear mixed-effects models (LMEMs), incorporating replicate and larvae ID (for individual behavior) as random factors and time/treatment as fixed effects. T-independent tests were used to determine significant differences between treatments at specific timeframes. For cardiorespiratory data, the non-parametric Kruskal–Wallis test was utilized to identify significant differences among independent groups. Each ANOVA was followed by a post-hoc Tukey’s HSD test, with significance set at *p* < 0.05. Hierarchical cluster analysis (HCA) identified treatments with comparable effects on fish, utilizing normalized data, Euclidean distance, and average linkage.

Statistical analyses and visualizations utilized STATISTICA (v10.0, StatSoft Innovations; Tulsa, OK, USA) and PRISM (v8.0.0, GraphPad Technologies; San Diego, CA, USA) software.

## 3. Results

### 3.1. The Short-Term Effects of GO on S. trutta Larvae

#### 3.1.1. Cardiorespiratory Functions

**Figure 3:**
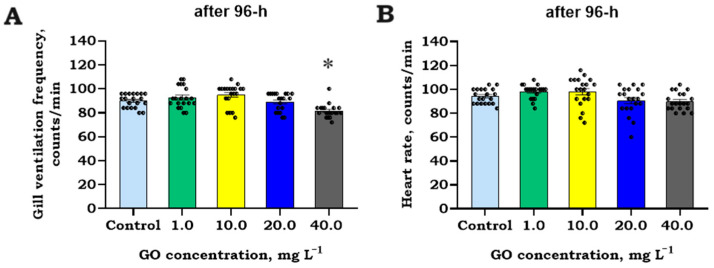
Gill ventilation frequency (GVF) (count/min), (**A**) and heart rate (HR) (count/min), (**B**) (mean ± SEM, raw data, *n* = 20) during 96 h exposure trials exposure to different concentrations of graphene oxide (GO). Asterisks (*) denote significant differences between treatments compared to the control level at significance levels of *p* < 0.05 (*).

The examination of cardiorespiratory endpoints of brown trout (*S. trutta*) larvae after 96 h revealed that only GVF showed any slight changes, with a 9.3 ± 2.1% decrease at the highest tested concentration (Kruskal–Wallis H = 27.3, *p* = 0.02; Figure 3A). In contrast, heart rate (HR) (Figure 3B) proved to be a less sensitive endpoint, displaying insignificant changes (*p* > 0.05) compared to the control level, ranging from 84 to 104 counts/min, similar to those exposed to GO concentrations at 20.0 and 40.0 mg L^−1^.

#### 3.1.2. Nesting Behavior

**Figure 4:**
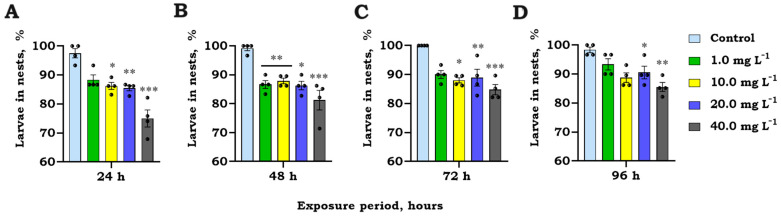
Number of brown trout (*S. trutta*) larval individuals (1 day after hatching) in nests (%) (**A**) (mean ± SEM, raw data, *n* = 4 repl.) after 24 h (**A**), 48 h (**B**), 72 h (**C**), and 96 h (**D**) exposure to different concentrations of graphene oxide (GO). Asterisks (*) denote significant differences between treatments compared to the control observational timeframe at significance levels of *p* < 0.05 (*), <0.01 (**), and <0.001 (***).

The nesting behavior of *S. trutta* larvae (Figure 4) was significantly impacted by GO over a 96 h period, revealing a noteworthy interaction between time and treatment (Repeated Measures ANOVA, F_12,45_ = 2.2, *p* = 0.031; Figure 4A). Following a 24 h exposure to GO concentrations of 10 (Tukey’s HSD test, *p* = 0.025), 20 (*p* = 0.002), and 40 (*p* < 0.001) mg L^−1^, a significant decrease (% change ± SEM), ranging from 10.8 ± 1.9 to 23.1 ± 4.3% of larvae in nests, was observed compared to the control level. Interestingly, as the length of GO exposure grew, the number of larvae in nests also grew, indicating a possible adaptive response to extended GO exposure.

### 3.2. The Short-Term Effects of GO on S. trutta Juveniles

#### Swimming Activity

**Figure 5:**
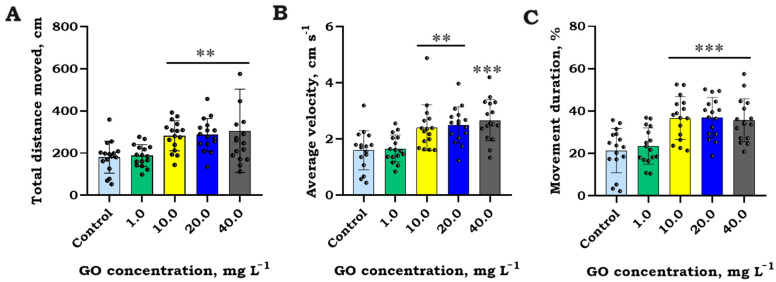
Total distance moved (cm) (**A**), average velocity (cm/s) (**B**), and (cumulative) movement duration (%) (**C**) (mean ± SEM, raw data, *n* = 16) for brown trout (*S. trutta*) juveniles after 2 h of exposure to different concentrations of graphene oxide (GO). Asterisks denote significant differences between treatments compared to the control observational timeframe at significance levels of *p* < 0.01 (**), and < 0.001 (***).

The obtained behavioral data in brown trout (*S. trutta*) juveniles revealed significant alterations in swimming activity endpoints, including total distance moved (F_4,75_ = 8.3, *p* < 0.001), average velocity (F_4,75_ = 9.2, *p* < 0.001), and movement duration (F_4,75_ = 7.9, *p* < 0.001), following a 2 h exposure to various concentrations of GO as illustrated in Figure 5. Analyzing these endpoints, the impact of GO on fish juveniles was characterized by a considerable increase from the control level, ranging from 53.5 ± 12 to 72.2 ± 15% (*p* < 0.01), across almost all tested concentrations (10.0, 20.0, and 40.0 mg L^−1^) of GO.

### 3.3. The Hierarchical Cluster Analysis

**Figure 6:**
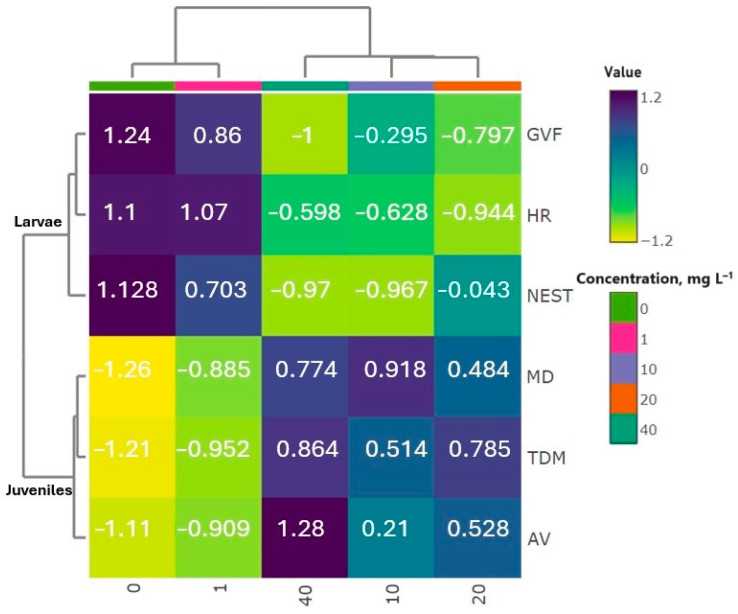
Hierarchical cluster analysis of brown trout (*S. trutta*) larvae and early juveniles exposed to different concentrations of GO based on the Euclidean distance coefficient and the average linkage method. Abbreviations: AV—average velocity (cm s^−1^), TDM—total distance moved (cm), MD—movement (cumulative) duration (%), HR—heart rate (counts/min), GVF—gill ventilation frequency (counts/min), and NEST—larvae in nests (%).

The hierarchical cluster analysis (HCA) revealed distinct groupings among the studied fish (brown trout) (Figure 6). The first cluster included *S. trutta* larvae and juveniles exposed to 1.0 mg L^−1^ GO, along with the control group, indicating similar behavioral responses. In the second major cluster, fish treated with 10.0 and 20.0 mg L^−1^ GO formed one subcluster, while those exposed to 40.0 mg L^−1^ GO comprised another. This detailed categorization highlights nuanced variations in fish responses to different GO concentrations, emphasizing concentration-dependent effects and the significance of dosage levels in shaping observed behavioral patterns.

### 3.4. The Effects of Graphene (Nanoflakes) on O. mykiss Larvae

#### Swimming Activity

**Figure 7:**
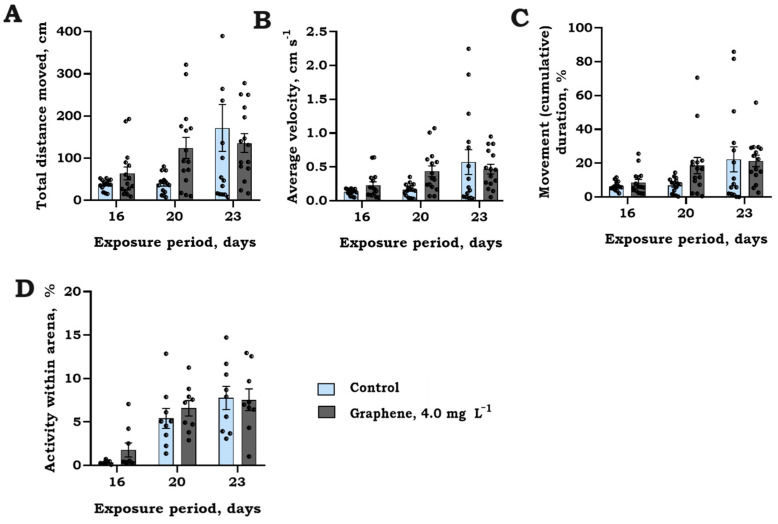
Total distance moved (cm) (**A**), average velocity (cm s^−1^) (**B**), movement (cumulative) duration (%) (**C**) (mean ± SEM, raw data, *n* = 15), and activity within arena (%) (**D**) (mean ± SEM, *n* = 9 repl.) of rainbow trout (*O. mykiss*) larvae after selected time intervals (16, 20, and 23 days) of exposure to graphene (nanoflakes) at a concentration of 4.0 mg L^−1^.

**Figure 8:**
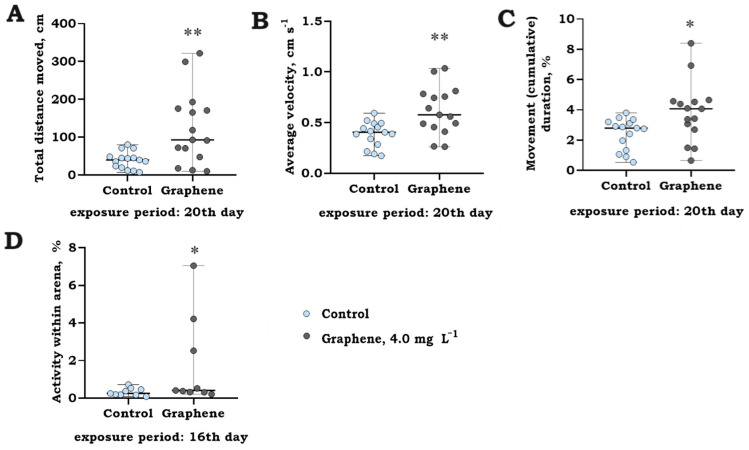
Total distance moved (cm) (**A**), average velocity (cm s^−1^) (**B**), movement (cumulative) duration (%) (**C**) (median with range, raw data, *n* = 15), and activity within arena (%) (**D**) (median with range, raw data, *n* = 9 repl.) of rainbow trout (*O. mykiss*) larvae following 16, 20, and 23 days of exposure to graphene (nanoflakes) at a concentration of 4.0 mg L^−1^. Asterisks denote significant differences between treatments compared to the control level at significance levels of *p* < 0.05 (*) and <0.01 (**).

Prolonged exposure of rainbow trout (*O. mykiss*) larvae to graphene (nanoflakes) at a concentration of 4.0 mg L^−1^ for 23 days did not reveal any significant interaction between time and treatment, as illustrated in Figure 7. A notable variability in individual responses was observed with an increasing duration of exposure. Nevertheless, when assessing variations in responses during specific observational timeframes, t-independent test analysis revealed significant differences in larval swimming activity, as indicated in Figure 8.

The presence of graphene nanoflakes resulted in noticeable behavioral changes on the 20th day of the exposure period, characterized by an increase in total distance moved (*t*-test = −3.27, *p* = 0.002; Figure 8A), average velocity (*t*-test = −3.38; *p* = 0.002; Figure 8B), movement (cumulative) duration (*t*-test = −2.44; *p* = 0.021; Figure 8C), as well as changes in activity within the arena following larvae exposure in a group, where activity was determined to be significantly increased (*t*-test= −2.19; *p* = 0.044; Figure 8D). Furthermore, it should be noted that during long-term exposure to graphene, larval activity significantly increased over development, contributing to the observed high variation in responses.

## 4. Discussion

This study aimed to investigate the impact of short-term (up to 96 h) and long-term (up to 23 days) exposure to graphene, in the form of nanoflakes, and its oxidized variant, graphene oxide (GO), on the early developmental stages and juveniles of two common salmonid species: brown trout (*S. trutta*) and laboratory-reared rainbow trout (*O. mykiss*). This research aimed to assess the effects of these carbon nanomaterials at both physiological and whole-organismal levels, providing a deeper understanding of the early life behavioral traits of freshwater fish that could impact their survival rates.

In the present study, exposure of *S. trutta* larvae to GO resulted in a reduction in nesting activity by 11–23%. Ordinarily, after hatching, larvae exhibit a natural behavioral trait of forming nests, driven by an instinct to grow in gravel spawning nests for protection. When fish experience stress, their natural response is to avoid and escape, leading to a lack of nest-building and, consequently, a decreased likelihood of survival. In addition to avoidance, alternative mechanisms may be implicated, such as the secretion of epidermal mucus as a defense against adverse environmental conditions and the uptake of nanoparticles [32]. In our long-term exposure study, we observed a significant increase (up to 3.3-fold) in the swimming activity endpoints of *O. mykiss* larvae when exposed to graphene nanoflakes. As swimming behaviors are closely linked to their cardiorespiratory systems, playing a crucial role in efficient oxygen uptake [33], it was noted that cardiorespiratory functions were also impacted by GO in *S. trutta* larvae, particularly at the highest tested concentration.

Despite the significant interest among scientists in carbon nanomaterials, toxicity tests have primarily focused on a variety of model organisms, with particular attention given to zebrafish (*Danio rerio*) during their early life stages. These studies aimed to explore the negative effects of graphene-based nanomaterials on physiology, histopathology, and behavioral patterns of the whole organism [19,34,35]. The impact of graphene derivatives on the swimming performance of both fish larvae and adult fish has been demonstrated to exhibit diverse effects, including increased or decreased activity, abnormal swimming patterns, and, in some cases, no discernible effects. Due to the adverse impact of graphene derivatives on fish behavior, several studies have proposed a potential association between these carbon nanomaterials and developmental neurotoxicity as well as immunotoxicity during the early developmental stages [9,14,30,36]. Moreover, changes in fish swimming activity have been associated with variations in acetylcholine esterase and cortisol levels following treatment with the oxidized form of graphene. This impact was determined in zebrafish larvae exposed to relatively low concentrations (0.1 and 1.0 mg L^−1^), similar to our lowest tested concentration [34]. Furthermore, the accumulation of graphene nanoparticles in the area of the head has been identified as a factor causing cellular damage in the brain, leading to degeneration and the production of autophagosomes, ultimately influencing behavior [30]. The available evidence suggests that the effects seen in fish are largely contingent on the duration of exposure, with a direct correlation to the dose and lateral size of these carbon compounds [15]. Despite various mechanisms proposed to explain changes in the behavior of fish larvae linked to the mode of action of graphene oxide, research on these nanoparticles neurotoxic effects is being extensively investigated.

Numerous investigations have primarily concentrated on evaluating developmental neurodegeneration and immune system damage caused by GO, with a primary focus on zebrafish embryos. However, it is crucial to consider and investigate the impact of GO on later developmental stages of fish. Early life fish juveniles are known to be sensitive, particularly salmonids, similar to larvae, to adverse environmental changes, including water pollution with nanocarbons. In addition to studying fish larvae, we explored the effects of GO on *S. trutta* juveniles at the age of 0+. Our study observed that *S. trutta* juveniles exposed to GO particles exhibited a substantial increase in swimming activity by 54–72% within first two hours, resembling the effects observed in *O. mykiss* larvae during long-term exposure. The adverse effects of GO on fish can be attributed to the mechanical harm inflicted on specific tissues and organs tailored to fish.

In the early stages of development, fish gills, being delicate organs and the primary point of entry for noxious substances, are susceptible to damage even in minimal concentrations. Graphene-based nanomaterials (GBNs) can cause irritation and damage to the gills by interacting with their branchial membrane or mucoproteins. This interaction may lead to acute gill irritation and a prompt avoidance response in fish, especially during the initial hours of exposure [37]. Consistent with the findings of a previous study [13], necrotic cell areas were identified in the gill tissues of zebrafish exposed to GO concentrations (10–20 mg L^−1^, respectively), mirroring the concentrations investigated in our research. On the other hand, some authors have suggested that hyperactivity might be related to the particles themselves, as particle aggregates form over time. Fish might chase these aggregates, which could be considered a feeding behavior [38]. Consequently, the potential effects of GO may be linked to particle size, exposure duration, and the underlying processes of the harmful action of these chemicals on specific target organs. These effects depend on the fish’s stage of development and may result in diverse biological responses, including those at a whole-organismal level. Despite numerous approaches to explaining alterations in the behavior of fish embryos and larvae attributed to the mode of action of graphene-based nanomaterials (GBNs), this area of research is still ongoing.

Existing limitations in GBN extraction from water and sediments impede precise assessments in aquatic environments. For a thorough prediction of GBNs’ adverse effects, future studies should consider nanomaterial type, characteristics, water behavior, potential concentrations, and various environmental abiotic factors influencing aquatic life. Analysis of the literature suggests environmental concentrations of various carbon nanomaterials may range from 0.1 up to 1000 μg L^−1^ [17,39], serving as an initial point for nanocarbon ecotoxicological analysis. As nanomaterial production advances across scientific domains, concentrations are likely to increase, underscoring the necessity for comprehensive studies on GBNs’ toxicity across diverse environmental contexts for both aquatic and terrestrial species.

## 5. Conclusions

This study examined the effects of two common carbon nanomaterials, graphene (nanoflakes) and its oxidized form, graphene oxide (GO), on early life salmonids (*S. trutta*, *O. mykiss*). The investigation explored both short- (up to 96 h) and long-term (up to 23 days) impacts on fish nesting behavior, combined with cardiorespiratory responses, and swimming performance. The results revealed that a short-term exposure to GO significantly influenced the behavioral responses of *S. trutta* larvae, causing an 11–23% reduction in nesting activity and a 54–72% increase in swimming activity among fish juveniles. Examination of cardiorespiratory endpoints in *S. trutta* juveniles showed a 9% decrease in gill ventilation frequency of larvae indicating physiological changes. Notably, graphene (nanoflakes) had a prolonged impact on *O. mykiss* larvae, leading to an increased level of fish swimming activity over a 20-day exposure period. The study concluded that GO exhibits concentration-dependent effects on the studied fish.

This study shows that studied carbon nanomaterials significantly affect fish nesting and swimming behavior in early development, potentially impacting population dynamics and individual survival in their natural habitats. Additional research is essential to grasp the safety profile of various carbon nanomaterials and their potential toxicity for diverse organisms. This includes a broader examination of biological responses in conjunction with the dynamic nature of the environment.

## Data Availability

The data presented in this study are available on request from the corresponding author.
